# Interactive Impacts of Beneficial Microbes and Si-Zn Nanocomposite on Growth and Productivity of Soybean Subjected to Water Deficit under Salt-Affected Soil Conditions

**DOI:** 10.3390/plants10071396

**Published:** 2021-07-08

**Authors:** Hany S. Osman, Salah M. Gowayed, Mohssen Elbagory, Alaa El-Dein Omara, Ahmed M. Abd El-Monem, Usama A. Abd El-Razek, Emad M. Hafez

**Affiliations:** 1 Department of Agricultural Botany, Faculty of Agriculture, Ain Shams University, Hadayek Shubra, Cairo 11241, Egypt; hany_osman1@agr.asu.edu.eg; 2 Department of Botany, Faculty of Agriculture, Suez Canal University, Ismailia 41522, Egypt; salahgowed@yahoo.com; 3 Department of Biology, Faculty of Science and Arts, King Khalid University, Mohail Assir 61321, Saudi Arabia; mhmohammad@kku.edu.sa; 4 Department of Microbiology, Soils, Water and Environment Research Institute, Agricultural Research Center, Giza 12112, Egypt; alaa.omara@yahoo.com; 5 Department of Agronomy, Faculty of Agriculture, New Valley University, New Valley, Elkharrga 72511, Egypt; abdelmonem07@agr.nvu.edu.eg; 6 Agronomy Department, Faculty of Agriculture, Tanta University, Tanta 31527, Egypt; usama.eldesouky@agr.tanta.edu.eg; 7 Department of Agronomy, Faculty of Agriculture, Kafrelsheikh University, Kafr El-Sheikh 33516, Egypt

**Keywords:** *Glycine max*, PGPR, nano-minerals, osmolyte, photosynthesis, plant water relations, antioxidant defense system, seed quality

## Abstract

Water stress or soil salinity is considered the major environmental factor affecting plant growth. When both challenges are present, the soil becomes infertile, limiting plant productivity. In this work a field experiment was conducted during the summer 2019 and 2020 seasons to evaluate whether plant growth-promoting microbes (PGPMs) and nanoparticles (Si-ZnNPs) have the potential to maintain soybean growth, productivity, and seed quality under different watering intervals (every 11 (IW_0_), 15 (IW_1_) and 19 (IW_2_) days) in salt-affected soil. The most extended watering intervals (IW_1_ and IW_2_) caused significant increases in Na^+^ content, and oxidative damage indicators (malondialdehyde (MDA) and electrolyte leakage (EL%)), which led to significant reductions in soybean relative water content (RWC), stomatal conductance, leaf K^+^, photosynthetic pigments, soluble protein. Subsequently reduced the vegetative growth (root length, nodules dry weight, and total leaves area) and seeds yield. However, there was an enhancement in the antioxidants defense system (enzymatic and non-enzymatic antioxidant). The individual application of PGPMs or Si-ZnNPs significantly improved leaf K^+^ content, photosynthetic pigments, RWC, stomatal conductance, total soluble sugars (TSS), CAT, POD, SOD, number of pods plant^−1^, and seed yield through decreasing the leaf Na^+^ content, MDA, and EL%. The combined application of PGPMs and Si-ZnNPs minimized the adverse impact of water stress and soil salinity by maximizing the root length, heavier nodules dry weight, leaves area, TSS and the activity of antioxidant enzymes, which resulted in higher soybean growth and productivity, which suggests their use under harsh growing conditions.

## 1. Introduction

Globally, salt-affected soils are one of the most serious ecological problems, particularly in arid and semi-arid regions [1], and are expected to exacerbate further in the coming era, as they have become a primary factor in reducing field crop productivity and negatively impacting sustainable agricultural development [2]. Around 25% of arable agricultural soils worldwide are affected by salt stress, and an estimated 1.5 million hectares are lost to production each year as a result of salt stress [3]. Soil salinization has a number of unintended consequences, including a decrease in the ability of plant roots to absorb water and nutrients [4,5], a decrease in morpho-physiological characteristics, and degradation of chlorophylls as a result of hyperosmotic and hyper-ionic effects resulting in oxidative damage [6,7,8,9]. Drought or deficit irrigation is another major adverse abiotic stress that affects plant development. Water stress, like salinity stress, simulates physiological dehydration and results in low water potential and osmotic stress [10]. Water stress has a detrimental effect on plant development and yield, resulting in a cascade of morphological, biochemical, and physiological disturbances [11,12], particularly in arid and semi-arid zones, where evapotranspiration exceeds the annual precipitation, which results in an increase in salt levels in the near-surface soil layer [13]. As a result, water stress interacts with soil salinity, both of which have a negative impact on plant performance, and development, resulting in loss the plant yield and quality [14].

The Leguminosae is the second largest plant family in the agriculture sector, and are considered as the most important source of vegetable protein in human diets and livestock forages, as well as ensuring long-term sustainable agriculture [15]. Soybean (*Glycine max* L.) is the most important cultivated legume cash crop rich in protein, carbohydrates, mineral nutrients, and oil [16]. Unlike the majority of legume crops, which are considered plants sensitive to osmotic stress, soybean has a little higher tolerance level which is regarded as a moderate sensitivity to salt stress [17,18]. The soil salinity threshold for soybean yield is 5.0 dS m^−1^, so a 50% reduction in soybean yield was observed with exposure to 7.5 dS m^−1^ soil salinity [18]. In contrast, drought stress could trigger a 24:50% reduction in the soybean seed yield [19]. Throughout the vegetative growth, numerous amino acids and sugars are promptly synthesized and transported during the reproductive growth to seeds. Thus, the development of soybean needs good agronomic practices for the increment of seed crop as the flowering and seed filling stages are the most drought-susceptible stages [20].

Remarkable studies have been conducted on the growth and productivity of crops grown under drought and salinity stress, and significant applications have been evaluated for their ability in improving the growth and development of field crops cultivated under drought and salinity stresses, which consider as an innovative approach for sustainable agriculture [21,22,23,24,25]. Beneficial microorganisms in the rhizosphere have positive effects on plant growth and development. Mycorrhizal fungi, N_2_-fixing bacteria, and plant growth-promoting rhizobacteria (PGPR) are the most rhizosphere species that have been extensively studied for their beneficial effects on plant growth and served as plant growth-promoting microbes (PGPMs) [26], which could limit the adverse impact of environmental stressors as well as are eco-friendly and low cost-efficient applications [27]. PGPMs are considered one of the important keys to solving environmental problems such as drought and salinity, which adverse osmotic stress and stimulate plant growth and development [28]. Application of PGPM could alleviate the negative impacts of abiotic stresses through the production of phytohormones, i.e., auxins, gibberellins, cytokinins, abscisic acid, as well as jasmonic acid, and salicylic acid, which has the ability to stimulate the systemic tolerance, in addition to producing exopolysaccharides (EPS) [28,29]. The synthesized phytohormones by PGPMs promote plant cell division and root length, which indirectly enhance the water absorption ratio and regulate the stomatal closure, osmolytes content, and improving activity of antioxidant enzymes, which cause an inhibition in the stress-related oxidative damages resulting in declined leaf transpiration which positively affecting seed production in plants subjected to different abiotic stresses [30].

*Bradyrhizobium japonicum* is a highly efficient nitrogen fixer that forms a symbiotic relationship with soybean. Under various environmental conditions, isolates from stress-affected soil are the most successful PGPMs [31]. Soybean co-inoculation with PGPMs and its natural symbiont (*B. japonicum*) altered plant growth parameters and markedly increased nodulation, and significantly increase the fixed-N_2_ amounts, which increase grain yield, thereby reducing reliance on fertilization with inorganic N [32,33]. *Trichoderma* is a genus present in many habitats, where certain strains have the potential to mitigate the severity of plant diseases by inhibiting the pathogens due to their high antagonistic potential [34]. When plants inoculated with *Trichoderma harzianum* are exposed to abiotic stress, *T. harzianum* alters the biosynthesis of plant secondary metabolites such as phytohormones and the osmolytes, which increases photosynthetic pigments, proline, soluble proteins, and vegetative growth, in addition to increasing the level of phenols, flavonoids, and IAA in the stressed plants [35,36].

A large number of proteins are contained or bound to Zn, which is the only metal used in all six enzyme groups, including oxidoreductases, transferases, hydrolases, lyases, isomerases, and ligases [37]. Plants grown under salinity or drought stress benefit from the application of Zn by increasing their tolerance level, which is reflected in enhanced plant growth, chlorophyll content, and biomass in rice [38], decreased proportion of aborted seeds per pod, and increased the yield quantity of pea [39], upregulation of the activity of antioxidant enzymes, and accumulation of osmolytes [40]. Silicon (Si) is a critical component of crop production, especially in minimizing the negative impacts of oxidative, salinity, and drought stresses [41,42]. Treating plants with nano-Si enhances the plant nutrition status by limiting the transportation of Na^+^ ions from roots to leaves and by increasing the level of K^+^ ions in leaves under soil salinity conditions [43]. It was recently discovered that plants treated with nanominerals as nutrients and stimulants showed improved physiological and biochemical attributes, decreased oxidative damage, stimulated water and nutrient uptake and improved plant growth, resulting in increased seed yields [44].

Previous studies have produced insufficiently reliable outcomes. There is an urgent need to investigate environmentally sustainable applications to improve soybean production under stress condition. To fulfil this goal, this study aimed to quantify the effects of foliar spray with nanominerals (Si-Zn) in combination with the soil application of PGPMs on the vegetative growth, plant water relation, antioxidant defense system, and seed yield of soybean plants cultivated in salt-affected soil under different watering intervals.

## 2. Results

A field experiment was conducted on soybean plants grown in salt-affected soil under different irrigation intervals (**IW_0_**; 11 days, **IW_1_**; 15 days, and **IW_2_**; 19 days) during two successive summer seasons (2019 and 2020). In contrast to well-watered soybean plants, all of the studied parameters were markedly reduced as a result of exposure to more extended irrigation periods under salt-affected soil. As a result of individual application of plant growth-promoting microbes (PGPMs) or nanocomposite (Si+Zn) and their combination, various variables such as exchangeable Na in the soil, plant biochemical and physiological characteristics have positively influenced the yield and related traits as well as seed quality. Regardless of irrigation intervals, the combined application of PGPMs and Si-ZnNPs resulted in a greater increase in all studied parameters compared to untreated plants (control).

### 2.1. Exchangeable Sodium Percentage (ESP)

**Figure 1:**
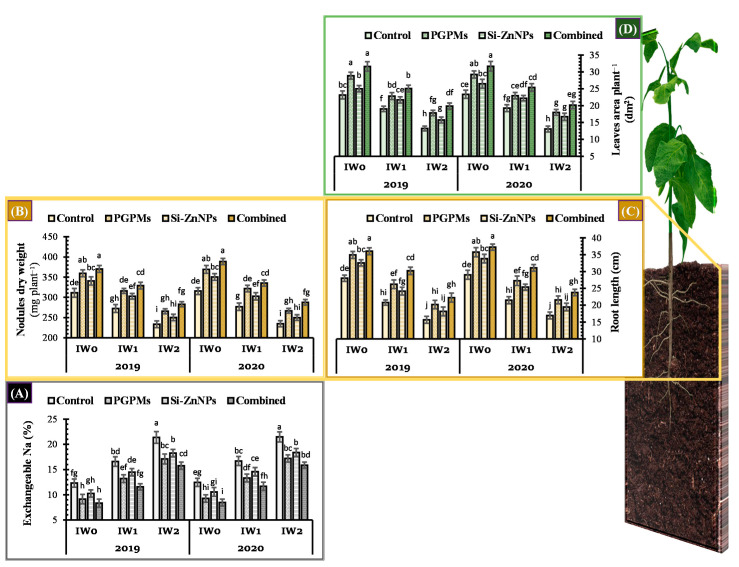
Influence of application of PGPMs, Si-ZnNPs and their combination on (**A**) the percentage of exchangeable Na in the soil, and (**B**) nodules dry weight, (**C**) root length, and (**D**) total leaves area of soybean plant grown in salt-affected soil with different levels of watering intervals (**IW_0_**; 11 days, **IW_1_**; 15 days, and **IW_2_**; 19 days) during the successive 2019 and 2020 growing seasons. The data presented as means ± SD. Means labeled with the same lower-case letter are not significantly different according to Duncan’s Multiple Range Test.

Comparing to well-watered plants (IW_0_), the longer watering intervals (IW_1_ and IW_2_) significantly increased the exchangeable Na percentage (ESP) in salt-affected soil (Figure 1A). On the other hand, ESP decreased marginally when Si-ZnNPs was applied on foliage, and it decreased even further when PGPMs were applied to the soil. In salt-affected soil, the greatest reduction in ESP was achieved by using a combination of PGPMs and Si-ZnNPs in both seasons and at various irrigation water intervals (Figure 1A). In both seasons, exposing soybean plants to a combination of PGPMs and Si-ZnNPs in IW_1_ restored the ESP in the soil as its percentage in the control treatment of the well watering condition (IW_0_). Similar results were obtained for the coupled treatment in IW_2_ compared to the control treatment under 15 days of interval watering (IW_1_).

### 2.2. Vegetative Growth Characteristics

#### 2.2.1. Root Length and Nodules Dry Weight

Longer irrigation cycles (IW_1_ and IW_2_) significantly decreased root length and nodules dry weight of soybean plants grown in salt-affected soil relative to well water plants (IW_0_) (Figure 1B,C). However, foliar application of Si-ZnNPs resulted in a slight significant increase in root length and nodule dry weight, while application of PGPMs resulted in a more substantial increase. The highest increase was observed when combined PGPMs and Si-ZnNPs were applied in both seasons, regardless of irrigation regime. According to the data provided in Figure 1, soybean plants subjected to IW_1_ treatment (irrigation every 15 days) with the application of combined PGPMs and Si-ZnNPs had longer roots and heavier nodules than control plants in IW_0_ (irrigation every 11 days). Similarly, soybean plants that received the IW_2_ treatment (irrigation every 19 days) with mixed PGPMs and Si-ZnNPs application had longer roots and heavier nodules than control plants in the IW_1_ treatment (irrigation every 15 days). Furthermore, the individual application of PGPMs surpassed Si-ZnNPs application in terms of root length and nodule dry weight in both seasons (Figure 1B,C).

#### 2.2.2. Total Leaves Area

Irrigation every 15 and 19 days significantly reduced the leaves area of soybean plants. However, under recommended irrigation (IW_0_), the foliar application of Si-ZnNPs recorded only a 7.9% increase in leaves area plant^−1^. In contrast, individual PGPMs application presented a higher effect, which increased the leaves area by 24.6% over control (Figure 1D). The highest increase percentage (36.3%) observed with the combined application PGPMs and Si-ZnNPs under IW_0_ treatment. Soybean plants irrigated every 15 days with a combination of PGPMs and Si-ZnNPs had a tiny higher leaves area plant^−1^ (8.3%) over control plants that irrigated every 11 days. Similarly, soybean plants watering every 19 days with mixed PGPMs and Si-ZnNPs application had only a 4.5% increase in the leaves area plant^−1^ over control plants that irrigated every 15 days (Figure 1D).

### 2.3. Concentration of Sodium and Potassium in Leaves

**Figure 2:**
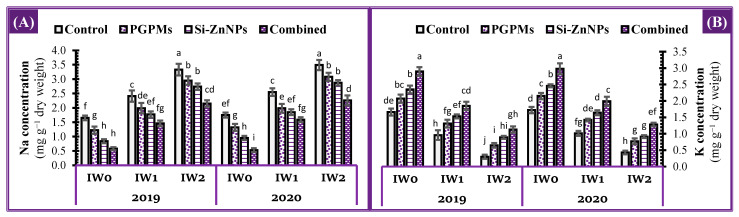
Influence of application of PGPMs, Si-ZnNPs and their combination on the concentration of (**A**) sodium (Na^+^), and (**B**) potassium (K^+^) in the leaves of soybean plant grown in salt-affected soil with different levels of watering intervals (**IW_0_**; 11 days, **IW_1_**; 15 days, and **IW_2_**; 19 days) during the successive 2019 and 2020 growing seasons. The data presented as means ± SD. Means labeled with the same lower-case letter are not significantly different according to Duncan’s Multiple Range Test.

As anticipated in salinized soils, longer watering intervals (IW_1_ and IW_2_) had a detrimental effect on the ion balance (low K^+^ and high Na^+^) in the leaves of soybean plants, resulting in decreased efficiency when compared to well water treatment (IW_0_) (Figure 2). Both IW_1_ and IW_2_ treatment reduced the content of K in leaves by 42.2% and 81.9% compared to IW_0_ control, meanwhile the content of Na increased by 46.1% and 101.8% over IW_0_ control treatment. Individual applications of PGPMs or Si-ZnNPs and their combination significantly affected K^+^ and Na^+^ content in the soybean leaves in salt-affected soil, irrespective of the watering interval applied (IW_0_, IW_1_, and IW_2_). The combined application reduced the Na^+^ levels by 64.8%, 39.4%, and 35.4% compared to the control of each treatment (IW_0_, IW_1_, and IW_2_). The efficiency of individual application Si-ZnNPs was higher than PGPMs on increasing K^+^ content and decreasing Na^+^ content in the leaves in both seasons compared to control plants. More significant increases in the leaf content of K^+^ were discovered as a result of the co-application (PGPMs and Si-ZnNPs), which increased by 74.5%, 92.7%, and 276.7% more than the control subjected to water stress at IW_0_, IW_1_, and IW_2_. Furthermore, soybean plants undergoing IW_1_ co-application treatment Si-ZnNPs and PGPMs increased K^+^ content by 11.4% and decreased Na^+^ content by 11.5% than control plants in IW_0_ (Figure 2).

### 2.4. Photosynthetic Pigments Concentration

**Figure 3:**
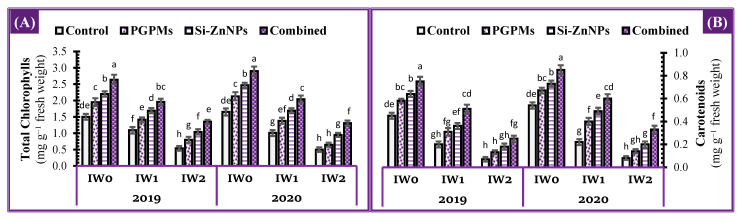
Influence of application of PGPMs, Si-ZnNPs and their combination on the concentration of (**A**) total chlorophylls, and (**B**) carotenoids in the leaves of soybean plant grown in salt-affected soil with different levels of watering intervals (**IW_0_**; 11 days, **IW_1_**; 15 days, and **IW_2_**; 19 days) during the successive 2019 and 2020 growing seasons. The data presented as means ± SD. Means labeled with the same lower-case letter are not significantly different according to Duncan’s Multiple Range Test.

Concentrations of photosynthetic pigments (chlorophyll and carotenoids) in the leaves of soybean plant were negatively affected by the interaction of longer intervals watering (IW_1_ and IW_2_) and the salt-affected soil compared to well water treatment (IW_0_), which markedly decreased the chlorophyll content by 27.3% and 64.0% in the control of IW_1_ and IW_2_ comparing to its content in IW_0_ (Figure 3). Furthermore, the addition of PGPMs or Si-ZnNPs alone or in combination reduced the adverse effects of IW_1_ and IW_2_ and improved the content of total chlorophyll and carotenoids. Under deficit water treatments (IW_1_ and IW_2_), plants inoculated with *B. japonicum* and *T. harzianum* in combination with nanoparticles (Si+Zn) showed the greatest increase in total chlorophyll (79.8%, and 150.0%) and carotenoids (155.0%, and 257.1%) over their own control. In contrast, comparing the combined treatment under IW_1_ and IW_2_ with the control of IW_0_, under interval watering every 15 days, the chlorophyll and carotenoids content increased by 30.7% and 13.3%, respectively, while under the longest interval watering (IW_2_), both pigments decreased by 10.0% and 44.4%, respectively. The minimum increment over the control treatment was noted for PGPMs sole application, while nanoparticles (Si+Zn) application was more effective than PGPMs regardless of irrigation intervals (Figure 3).

### 2.5. Osmoprotectants

**Figure 4:**
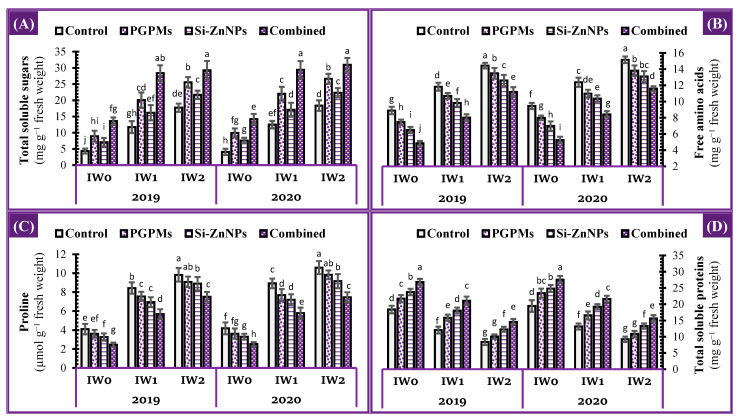
Influence of application of PGPMs, Si-ZnNPs and their combination on the concentration of (**A**) total soluble sugar, (**B**) free amino acids, (**C**) proline, and (**D**) total soluble proteins in the leaves of soybean plant grown in salt-affected soil with different levels of watering intervals (**IW_0_**; 11 days, **IW_1_**; 15 days, and **IW_2_**; 19 days) during the successive 2019 and 2020 growing seasons. The data presented as means ± SD. Means labeled with the same lower-case letter are not significantly different according to Duncan’s Multiple Range Test.

Changes in the biochemical parameters that have an osmo-protective role in the leaves of soybean plants cultivated under different irrigation intervals, i.e., total soluble sugars (TSS), proline, free amino acid (FAA), and soluble protein (SP), are presented in Figure 4. The prolonged intervals of watering (IW_1_ and IW_2_) resulted in substantial increases in the osmolytes content (TSS [171.2%, and 309.5%], FAA [33.0%, and 62.2%], and proline [104.7%, and 138.7%]) of soybean leaves, whereas significant decreases in the soluble proteins (34.2%, and 54.2%) were observed under the same watering conditions compared to controls of well-watered irrigation (IW_0_). In contrast, sole application of PGPMs under IW_0_, IW_1_, and IW_2_ resulted in a gradual decrease in leaf proline and FAA content, while gradual increases in SP and TSS were observed. Whereas the individual application of Si-ZnNPs under IW_0_, IW_1_, and IW_2_ lowered the content of proline, FAA, and TSS than PGPMs application, while a higher increase in SP content was observed comparing to PGPMs sole application. Treating soybean plants with the co-application of PGPMs and Si-ZnNPs under IW_0_, IW_1_ and IW_2_ showed the greatest decrease in the content of proline (40.3%, 32.5%, and 23.6%), FAA (45.3%, 32.2%, and 22.5%), as well as the greatest increase in the content of TSS (213.6%, 141.9%, and 65.0%) and SP (46.2%, 74.4%, and 73.7%) comparing to their controls. In comparison between the combined application (PGPMs and (Si-ZnNPs) under longer watering intervals and the control of well irrigation treatment (IW_0_), it revealed that TSS increased by 6-fold its content under both IW_1_ and IW_2_ conditions (Figure 4).

### 2.6. Activity of Antioxidant Enzymes

**Figure 5:**
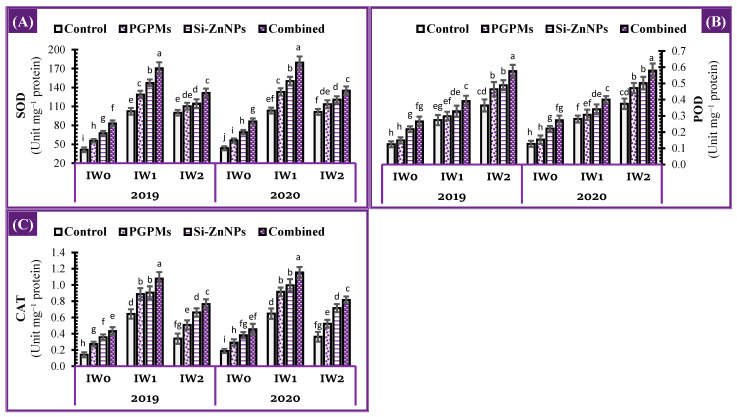
Influence of application of PGPMs, Si-ZnNPs and their combination on the activity of antioxidant enzymes, i.e., (**A**) SOD, (**B**) POD, and (**C**) CAT in the leaves of soybean plant grown in salt-affected soil with different levels of watering intervals (**IW_0_**; 11 days, **IW_1_**; 15 days, and **IW_2_**; 19 days) during the successive 2019 and 2020 growing seasons. The data presented as means ± SD. Means labeled with the same lower-case letter are not significantly different according to Duncan’s Multiple Range Test.

The activities of the antioxidant enzymes, i.e., superoxide dismutase (SOD), peroxidase (POD), and catalase (CAT) in the leaves of soybean plants grown under different watering intervals (11, 15, and 19 days), in regard to PGPMs and/or (Si+ZnPNs) applications are presented in Figure 5. In comparison to well water treatment (IW_0_), the association between longer intervals (IW_1_ and IW_2_) and soil salinity resulted in higher POD, SOD, and CAT. The most increased enzyme activity of SOD (147.3% and 141.5%) and CAT (353.3% and 140.0%) were observed under the IW_1_ condition, followed by the IW_2_ condition, comparing to the control of IW_0_ treatment. The individual application of Si-ZnNPs was superior to PGPMs in the activity of all tested antioxidant enzymes. The largest increases in the activity of CAT, POD, and SOD were observed as a result of foliar spraying with the nanocomposite (Si+Zn) in combination with soil application of PGPMs, which was more successful than their sole applications under the longest intervals (IW_1_ and IW_2_) conditions. SOD activity under the effect of combined application increased by 101.2%, 66.4%, and 31.5% under IW0, IW_1_, and IW_2_ over the controls of each treatment. Whereas the increased percentages in SOD under IW_1_ and IW_2_ in relevant to control of IW_0_ were much higher, 311.6% and 217.6%. The same trend was observed for CAT activity. Although SOD and CAT activity increased under the IW_1_ and IW_2_ conditions, their levels retreat under the IW_2_ condition. The gradual increases in enzyme activity under prolonged conditions were for the POD enzyme (Figure 5).

### 2.7. Oxidative Damage Status

#### 2.7.1. Lipid Peroxidation

**Figure 6:**
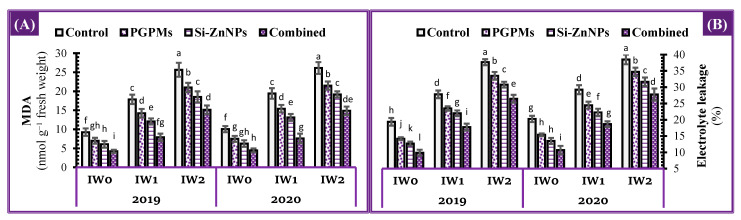
Influence of application of PGPMs, Si-ZnNPs and their combination on oxidative damage indicators, i.e., (**A**) malondialdehyde (MDA), and (**B**) electrolyte leakage (EL%) in the leaves of soybean plant grown in salt-affected soil with different levels of watering intervals (**IW_0_**; 11 days, **IW_1_**; 15 days, and **IW_2_**; 19 days) during the successive 2019 and 2020 growing seasons. The data presented as means ± SD. Means labeled with the same lower-case letter are not significantly different according to Duncan’s Multiple Range Test.

Malondialdehyde (MDA) concentration under different watering intervals (IW_0_, IW_1_, and IW_2_), as an indicator for the lipid peroxidation in leaves tissue of soybean plants, is shown in Figure 6. Exposing the control plants to the prolonged durations (IW_1_ and IW_2_) increased the levels of MDA by about 2-fold and 3-fold higher than its level under IW_0_. Foliar spray with nanocomposite (Si+Zn) decreased the MDA levels under all intervals more than PGPMs did. The superior application in reducing the MDA levels was the co-application with PGPMs and Si-ZnNPs. Compared to each irrigation treatment control, the combined application could reduce the MDA level by 54.3%, 55.9%, and 41.1% under IW_0_, IW_1,_ and IW_2_, respectively. The potency of combined application to control the MDA levels under prolonged intervals was much higher under IW_1_, which reduced MDA levels by 14.8% than control of IW_0_. In contrast, under IW_2_, the combined application could not lessen the MDA level below or equal its level under IW_0_, whereas, on the contrary, it recorded an increase of 63.4% over IW_0_ control treatment (Figure 6A).

#### 2.7.2. Electrolytes Leakage Percentage

In the 2019 and 2020 seasons, soybean plants that were irrigated at longer intervals (IW_1_ and IW_2_) and grown in salt-affected soil had a higher electrolyte leakage percentage (EL%) than plants that have will irrigation (IW_0_) (Figure 6B). The levels of EL% in the control plants increased by around 1.5-fold and 2-fold when exposed to the extended durations (IW_1_ and IW_2_) comparing to IW_0_ control treatment. Levels of EL% were lower in all intervals when foliar spray with nanocomposite (Si+Zn) was used instead of PGPMs, where both applications (PGPMs or Si-ZnNPs) recorded a significant reduction than their controls. Co-application with PGPMs and Si-ZnNPs was found to be the most effective in lowering EL% levels under all watering intervals levels. The combined application reduced EL% levels by 49.1%, 36.2%, and 29.7% under IW_0_, IW_1_, and IW_2_, respectively, as compared to the control of each watering treatment. The efficacy of co-application (PGPMs and nanocomposite (Si+Zn)) to regulate EL% level over longer interval was much higher under IW_1_, which decreased EL% by 8.4% compared to the control of IW_0_. The combined application under IW_2_ was unable to reduce EL% below its observed under IW_0_, resulting in a 36.6% increase over the IW_0_ control (Figure 6B).

### 2.8. Plant Water Relations

**Figure 7:**
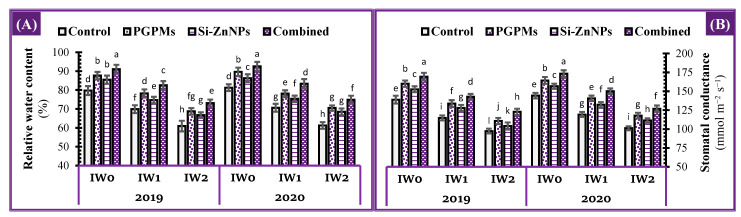
Influence of application of PGPMs, Si-ZnNPs and their combination on water relations, i.e., (**A**) leaf relative water content, and (**B**) stomatal conductance in the leaves of soybean plant grown in salt-affected soil with different levels of watering intervals (**IW_0_**; 11 days, **IW_1_**; 15 days, and **IW_2_**; 19 days) during the successive 2019 and 2020 growing seasons. The data presented as means ± SD. Means labeled with the same lower-case letter are not significantly different according to Duncan’s Multiple Range Test.

Soybean plants that were irrigated at longer intervals (IW_1_ and IW_2_) and grown in salt-affected soil had lower water relations characteristic in the plant (stomatal conductance (gs) and relative water content (RWC)) than plants that were well irrigated (IW_0_) (Figure 7). Under the mentioned conditions, for the controls of IW_1_ and IW_2_, RWC recorded a decrease by 12.3% and 23.6%, while gs recorded 17.4% and 29.9% decrease below control of IW_0_. 

Under IW_0_, IW_1_, and IW_2_ condition, RWC began to steadily improve over their controls as a result of individual application of PGPMs by 9.9%, 11.8%, and 12.8%, whereas nanocomposite (Si+Zn) had lower effects which increased by 7.3%, 6.9%, and 9.5%, respectively. The highest RWCs under all watering intervals (IW_0_, IW_1_, and IW_2_) were observed for the combined application PGPMs and Si-ZnNPs, which increased by 14.2%, 18.1%, and 19.9%, respectively. Although the highest percentage over its own control was recorded for combined application under the most extended watering interval (19 d), its positive effect over the threshold (control of IW_0_) was limited to IW_1_ (15 d) which increased by 3.6%, while under IW_2_ decreased the RWC by 8.3%. 

The same trend was observed for stomatal conductance (Figure 7). The co-application (PGPMs and Si-ZnNPs) under IW_0_, IW_1_, and IW_2_ comparing with their own controls increased the stomatal conductance by 22.0%, 24.4%, and 26.2%%, respectively, whereas when comparing with control of IW_0_ recorded an increase under IW_1_ by 2.7%, and a decrease under IW_2_ by 11.5% (Figure 7B).

### 2.9. Yield Related Traits and Seed Quality

The soybean yield-related traits such as the number of pods plant^−1^, 100-seed weight, and seed yield (t ha^−1^), as well as the seed nutritional quality such as the content of protein and carbohydrate, were negatively affected and significantly declined in soybean plants grown in salt-affected soil and subjected to the longer irrigation intervals (IW_1_ and IW_2_) compared to plants under IW_0_ condition during the successive 2019 and 2020 growing seasons (Table 1).

The yield-related traits were positively improved under individual applications of PGPMs or Si-ZnNPs, as well as their combination under all tested irrigation intervals (IW_0_, IW_1_, and IW_2_). Seed yield under well irrigation (IW_0_) was related to the type of application, where the sole application increased the seed yield by 19.1% for PGPMs and 24.8% for Si-ZnNPs. Additionally, as expected, the combined application (PGPMs and Si-ZnNPs) recorded the highest seed yield (35.3%) over the IW_0_ control. Meanwhile, under the extended watering intervals (IW_1_ and IW_2_), the seed yield of control decreased dramatically by 22.2% and 44.6% below the control of IW_0_. Application of nanocomposite (Si+Zn) was more effective than PGPMs in increasing the yield traits, which increased the seed yield by 24.8%, 23.8%, and 32.7%, compared to the controls of IW_0_, IW_1_, and IW_2_. The combined application under the same watering intervals recorded the highest seed yield, which increased by 35.3%, 39.7%, and 50.1%, over the controls of IW_0_, IW_1_, and IW_2_. On the other hand, the effect of combined application (PGPMs and Si-ZnNPs), when compared to the control of IW_0_, revealed that the only positive increase was under IW_1_ (8.6%), while under IW_2_, the seed yield decreased by 16.8% below the control of IW _0_. The same trends were observed for pods number per plant and 100 seeds weight. 

Seed content from proteins and carbohydrates was strongly related to 100 seed weight. The combined application increased the protein content by 36.2%, 63.5%, and 84.1%, and carbohydrates content by 38.1%, 40.1%, and 51.9%, compared to the controls of IW_0_, IW_1_, and IW_2_. The pronouncing of these greatest increase was revealed after comparing with the control of IW_0_, where the positive increases were only under IW_1_, which are 7.0% for proteins, and 9.9% for carbohydrates (Table 1).

## 3. Discussion

Deficit irrigation, drought, and soil salinity have the most important detrimental effects on the growth, productivity, and sustainability of crops, by affecting both plant and soil health. Osmotic stress (salinity or drought) negatively affects the root architecture and the ecosystem in cultivated soil, which reflects on soil chemical and physical traits, and indirectly connects with the mineral nutrition, water relations, antioxidant defense system, and metabolism of soybean, which reflect in turn on plant development, yield, and related traits. Nevertheless, little is known about the plant’s adaptive mechanisms. The current study has been investigated to evaluate the response of individual soil application of plant growth-promoting microbes or foliar spray of Si-Zn nanocomposite, and their mixture on mitigation the harmful effects of water deficit and soil salinity as well as improving the growth and development of cultivated soybean plants within deficit irrigation.

### 3.1. Yield Physiology under Well Irrigation

#### 3.1.1. Rhizosphere-Shoot Connections

Cultivation of the crops in salt-affected soil exhibits various negative impacts on plant aspects related to crop growth, which depend on soil salinity level, tolerance degree of the cultivated plant, and the availability of water in the soil. Under well-watering conditions, the deleterious effects of soil salinity on cultivated plants will be lessened to minimum levels. Plants can suffer from a variety of nutritional issues as a result of soil salinity. The abundance of a particular ion in soil solution, such as Na^+^ or Cl^−^, is thought to be the key cause of these nutrition problems. The presence of these soluble ions will reduce the availability of other essential minerals in the soil, resulting in decreased element accessibility and uptake by plants. Hence, treating soybean plants grown in salt-affected soil with PGPMs and/or Si-ZnNPs could boost the tolerance level to soil salinity, reflecting on maximizing the vegetative soybean growth (Figure 1).

Application of PGPMs (*B. japonicum* and *T. harzianum*) promoes the health of rhizosphere and plant, which minimizes the percentage of exchangeable Na (ESP) in the soil (Figure 1A), increase the solubility of essential minerals in the soil, and subsequently, increases their concentration in the shoot tissues, especially for K^+^ ions (Figure 2B). Enhancing the solubility, uptake and translocation of mineral nutrients in soybean by PGPMs application improved vegetative growth (Figure 1).

The *B. japonicum* (USDA 110) strain is capable of producing higher amounts of the growth-promoting phytohormones (IAA, GA_3_, and zeatin) in the growth medium [45]. Therefore, inoculation of soybean seeds with *B. japonicum* not only promotes plant growth by symbiotic N_2_ fixation but also has additional growth-promoting properties. The presence of IAA was linked to the stimulation of physiological and biochemical processes related to nodule formation and root development [45]. IAA raised the number of lateral roots and root hairs, increasing the plant–microbes interaction, which significantly affected nodule development and increased the nodules number [32]. Additionally, zeatin may have a role at the beginning of nodulation, while synthesized GA_3_ could be effective in promoting soybean shoot growth [45]. The genus *Trichoderma* is widely used as a biological control agent by inhibiting plant pathogens and promoting plant growth by producing different types of secondary metabolites [36]. Even under non-stressed condition, the application of *T. harzianum* could colonize with soybean roots, causing some anatomical changes such as increasing the thickness of the root (cortex, epidermis, and vascular cylinder) and also increasing the thickness of the mesophyll layer [46]. Therefore, inoculation of soybean seeds with the microbial consortium (*B. japonicum* and *T. harzianum*) magnified the growth-promoting effects, which subsequently reflecting in significant increases in root length, nodules, and total leaves area (Figure 1). The successful colonization with PGPMs increases the root exudates that directly affect soil biota activity and the efficiency of soil microbes in transforming the complex organic substrates and releasing the soil minerals, facilitating plant growth.

Foliar application of Si-Zn nanoparticles reduced the ESP in the soil and increased the nodules mass, root length, and total leaves area plant^−1^ (Figure 1). These positive effects could be related to the role of Zn in synthesizing some phytohormones like auxins. Zinc has an essential role in the biosynthesis of IAA from tryptophan [37], which responsible for nitrogen fixation, regulating the nodule formation, number and size of nodules [47]. Additionally, the application of Zn increased the content of endogenous GA_3_ [37], which alongside IAA, play an active role in cell enlargement, leading to elongating both of root and shoot system [47]. Moreover, Si has many well-documented beneficial effects on plant health. Si application to leguminous crops had the ability to increase the capacity of plant roots to release more isoflavonoids, which entice N_2_-fixing rhizobacteria [48], which is reflected by a greatly increase in the nodule size and number (Figure 1B). Additionally, soybeans treated with Si had a larger root diameter [48].

The combined soil and foliar application of PGPMs and Si-ZnNPs, respectively, boosted the performance of the soybean plant to its maximum capacity, which reduced the ESP in the soil to 32.5% lower than control, while nodule DW increased by 19.1%, root length by 28.6%, and leaves area plant^−1^ by 36.3% over control (Figure 1). These positive findings are in agreement with our previous work on faba bean [25], where the dual application of PGPR and silica recorded heavier nodule dry weight and root length.

#### 3.1.2. Physio-Biochemical Changes and Yield Relationship

The healthy vegetative growth of plants is a significant sign for the physiological functions, and their related assimilates follow the plant’s genetic codes written for optimum condition, so the application of any growth-promoting material will transfer the physiological processes to a new level and enhance the metabolism, which is reflected in a promotion of all growth stages and the better development of the plants. From this point of view, all the tested applications PGPMs and/or Si-ZnNPs increased all the studied physiological parameters, where the ultimate increases were for the foliar and soil combined application (PGPMs and Si-ZnNPs).

Under well irrigation conditions, the combined application significantly increased the photosynthetic pigments (Figure 3), total soluble sugars (TSS), total soluble proteins (TSP) while decreasing the osmolytes (free amino acids and proline) (Figure 4), while increasing the activity of antioxidant enzymes (Figure 5), which is definite reflected in a decrease of the oxidative damage in the form of lipid peroxidation and EL% to its minimum levels (Figure 6). The previous motivation in enzymatic and non-enzymatic antioxidants positively affects the photosynthetic machinery, increasing the assimilation rate and assimilates, which were related to the increases in stomatal conductance and RWC (Figure 7). 

The previously mentioned positive effects may result from the integration between every growth promoter used and the multi-functions of other tested promoters. Plant growth-promoting microbes (PGPMs) may establish themselves on the plant roots, stay there and compete with other microbes while promoting plant growth. Under normal conditions, *Trichoderma* can cause anatomical changes in different soybean organs and increases the stomatal index of the soybean plants [46]. Regarding mineral nutrition with nanoparticles (Si+Zn), zinc plays a variety of roles in plant development, pollen grain formation, and productivity, among other micronutrients. In legume crops, Zn requirements are higher during the reproductive stage than during the vegetative stage. If the amount of Zn available is insufficient, the photosynthesis process will be hampered by the altered structure of chloroplasts, resulting in reduced stem growth, smaller leaves, and later maturity [37]. Zn is involved in a variety of metabolic and enzymatic processes, as well as the production of growth hormones, which are essential for chlorophyll formation [39]. Since pollen grains contain a higher amount of Zn, the higher number of pods plant^−1^ and 100-seed weight may be due to the role of Zn in the reproductive process during fertilization [47]. The role of Zn in photosynthesis, N_2_ metabolism, IAA synthesis, and several other enzymatic reactions, where Zn acts as a cofactor [39], and thus influences plant growth and development, which could explain the increase in the number of seeds per pod and 100-seed weight. Additionally, Zn improved carbohydrate and protein synthesis as well as their transport to the developed seed, resulting in a larger source size [47]. Soybean plants treated with silicon displayed improvements in net photosynthesis, stomatal conductance (gs), and the content of intercellular CO_2_, even when plants were exposed to high salinity stress. This is because absorbed Si builds up in the leaves and stems’ cell walls, enabling the plant to start standing up more and increases the size usable for solar radiation and gas exchange, thereby increasing the performance of Photosystem II. Furthermore, Si accumulation enables stomata to open, which may increase transpiration rate and CO_2_ influx. From this perspective, Si treated leaves may be responsible for sustaining transpiration and enabling the crop to use the available water to achieve a higher net assimilation rate [48].

**Figure 8:**
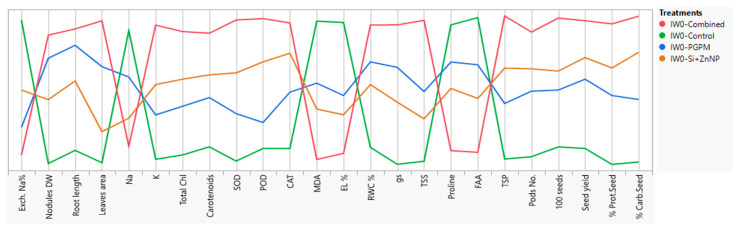
Parallel coordinates comparison plot showing how the studied variables of soybean grown under well irrigation changes in relation to the type of growth promoters (PGPMs and/or Si-ZnNPs).

Increasing the seed yield and all its related traits under the combined application (Table 1) revealed that these increases are correlated to enhancements that occurred to all previously mentioned parameters. But, which variable(s) was(were) more related to the increases in yield parameters. The answer to this question is extracted from the parallel coordinates’ comparison between all variables and their shares to other variables (Figure 8). The increases in yield-related traits were higher for the combined application (PGPMs and Si-ZnNPs) > Si-ZnNPs > PGPMs > control under IW_0_ conditions. Comparing all variables between combined application and Si-ZnNPs application reveal that the positive parallel increases were only for antioxidant enzymes (CAT, POD, and SOD) and osmolytes (FAA and proline) which protected the photosynthetic pigments (chlorophylls and carotenoids) and increased their efficiency, which directly and indirectly affected the physiological processes and increased the yield and its quality even under well-watering condition (Figure 8).

### 3.2. Yield Physiology under Deficit Irrigation

Cultivation in salt-affected soil is a risky practice, which needs more attention to other environmental factors. The most critical factor in this type of soil is the availability of water used for irrigation. Any tiny delay in irrigation by more than recommended duration in this specific area will expose the grown plants to different degrees from a combination of dual stress (water deficit and salinity stresses). Both of those stresses will initiate many signal transduction chains depending on the defense genome in the cultivated plant, reflected on multi-crosstalk between cells of root and shoot, and finally translate to multiple biochemical and physiological changes related mainly to plant survival under this type of stresses [49]. If the cultivated plant doesn’t have good defense genes, it will start suffering from the deleterious effects of these abiotic stresses, which is reflected in the plant growth, development, and productivity. Under this condition, exogenous support with growth promoters will add many benefits and extra defense mechanisms to the plants.

**Figure 9:**
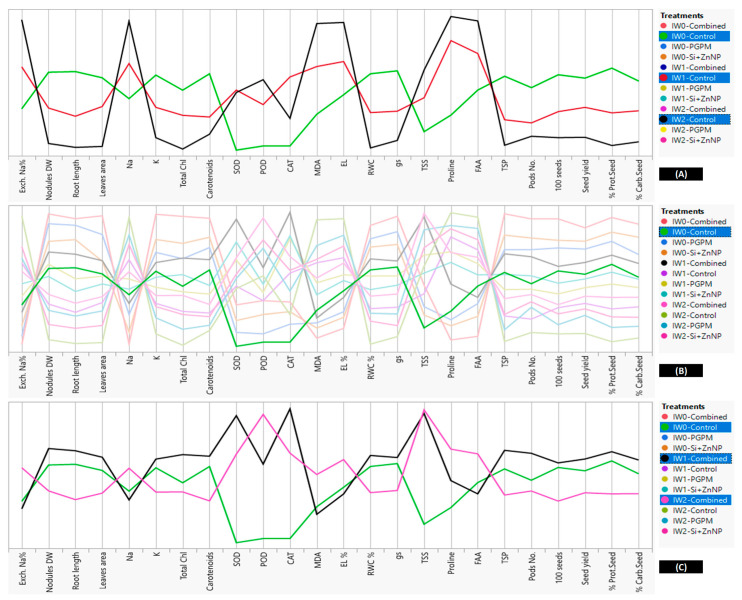
Parallel coordinates comparison plot showing how soybean studied variables changes in relation to the changes in irrigation levels, (**A**) comparison between controls under different intervals of watering, (**B**) comparison between all treatments in regard to the control of well watering as a reference, (**C**) comparison between the effect dual application of PGPMs and Si-ZnNPs under the higher levels of deficit irrigation in regard to control of well watering as a reference.

Subjected soybean plants to two levels of deficit irrigation (IW_1_ and IW_2_) resulted in significant reductions in many of variables (nodules D, root length, total leaves area, K, photosynthetic pigments, TSP, RWC, gs, and yield traits) and increases in other variables (ESP, Na, TSS, FAA, proline, SOD, POD, CAT, MDA, and EL%), comparing to the relative levels under IW_0_ (Figure 9A). A closer look at the parallel comparison between these variables (Figure 9A) under IW_2_ comparing to the level of change in IW_0_ and IW_1_ reveal that the most effective direct negative variables that started the deleterious events are ESP in the soil and Na^+^ content in the plant tissues which reflected on increasing the peroxidation levels and EL%. Unfortunately, although the content of osmolytes (FAA and proline) and activity of POD were much higher, they can’t mitigate enough the impact of water deficit, which resulted in the maximum recorded reduction in RWC and gs, leading to reduced carotenoids (antioxidant) content which is reflected by more inhibition and oxidation of chlorophylls and subsequently the assimilation rate and extremely inhibited the growth of root and shoot system.

Parallel comparison between the levels of variables in the control of IW_0_ (sharpen green line) in regard to all other applications (fade lines) under all watering treatments found that the position of most control parameters (sharpen green line, Figure 9B) laid on the middle between all other variables under IW_0_, IW_1_, and IW_2_ except for antioxidant enzymes (SOD, POD, and CAT) and TSS which recorded the lowest levels between all treatments (Figure 9B). This observation reveals that the most sensitive parameters related to defense mechanisms are the antioxidant enzymes and TSS.

Regarding the best-recommended application for motivating the growth and yield of soybean plant grown under different watering intervals, which is the co-application of (PGPMs and Si-ZnNPs), the parallel comparison for this combined treatment under the longer watering intervals (IW_1_ and IW_2_) is presented in Figure 9C. For more clarity, the comparison was made concerning the control of IW_0_ to reveal why the combined application under the most extended duration (IW_2_) was not enough to produce yield near enough from the control yield under IW_0_. Although motivated the TSS to its maximum limit under IW_2_ comparing to IW_1_, the antioxidant enzymes didn’t take this trend where the activity levels of SOD and CAT start to retreat under IW_2_. This retreat is more correlated to the highest increase in EL% and MDA, which indicated that cell membranes were suffering from severe oxidation, which has a direct correlation with decreasing the RWC, gs, and carotenoids, which in turn minimize the chlorophylls, plant growth and 100-seed weight (Figure 9C).

## 4. Materials and Methods

### 4.1. Experimental Layout

Two field trials were performed in 2019 and 2020 at Sakha Agricultural Research Station, Agricultural Research Center (ARC), Egypt, to assess the impacts of seed inoculation with a mixture of two microorganisms; (1) the bacteria *Bradyrhizobium japonicum* (USDA 110) and (2) the fungi *Trichoderma harzianum*, in addition to the foliar application of some beneficial and micro-elements in nanoparticles form on soil chemistry, plant vegetative growth, the activity of the antioxidant defense system, and related physiological attributes, yield and seed quality of soybean plants subjected to different irrigation intervals every 11 (IW_0_), 15 (IW_1_) and 19 (IW_2_) days at salt-affected soil. 

The experiment was laid out in a split-plot design with three replicates. The main plots were divided into irrigation intervals, and sub-plots were divided into the growth promoters’ treatments (Control, PGPMs, Si-ZnNPs, and the combination of PGPMs and Si-ZnNPs). The area of the sub-plot was 42 m^2,^ including 10 rows, 7 m long × 6 m wide. Soybean seeds (*Glycine max* L. cv. Giza 111) were obtained from Field Crops Research Institute, Department of Leguminous Crops, Sakha, Agricultural Research Station, Egypt. Seeds of soybean were planted on 28 May 2019 and 29 May 2020 at the rate of 100 kg ha^−1^. Plants were thinned to one plant per hill prior to first irrigation. Nitrogen fertilization was added as urea (46.5% N) at the rate of 18 kg N ha^−1^. Phosphorus fertilization was applied as calcium superphosphate 15.5% P_2_O_5_ at the rate of 80 kg P_2_O_5_ ha^−1^ prior to planting. Potassium fertilization applied at (114 kg K_2_O ha^−1^) before planting.

### 4.2. Soil Physicochemical Analysis

Soil samples from the experimental site (0–30 cm depth) were collected by an auger and air-dried to estimate the physicochemical properties, which are as following: The texture of the experimental site was clayey, consisting of 28.25 % sand, 24.37 % silt and 47.38 % clay with an initial pH of 8.23 (1:2.5 soil: water suspension) and an average exchange sodium percentage of 21.6%. Total N and organic C contents were 9.92 mg kg^−1^ and 11.55 g kg^−1^, respectively [50]. The electrical conductivity of saturated soil-paste extract (ECe) is 5.52 dS m^−1^.Soluble cations of Ca^++^, Mg^++^, Na^+^ and K^+^ were 9.43, 7.82, 37.34 and 0.38 meq L^−1^, respectively [51]. 

### 4.3. Treatments Preparation and Application

#### 4.3.1. Seed Inoculation with Microbial Consortium

Soybean seeds were sterilized with ethanol (70%) and Clorox (10%) for 3 min and bathed with autoclaved deionized water. Two microorganisms *Bradyrhizobium japonicum* (USDA 110) and *Trichoderma harzianum* were selected for their abilities to promote the growth of soybean [52]. Both strains were attained from Agricultural Microbiology Department, Soils, Water and Environment Research Institute (SWERI), Agricultural Research Centre (ARC), Egypt. Pure cultures of bacteria (*B. japonicum*) and fungi (*T. harzianum*) were routinely maintained on Yeast Extract Mannitol Broth (YEMB) medium and Potato Dextrose Broth (PDB) medium [53], respectively. The inoculation was prepared as peat-based inoculum, 10 mL of *B. japonicum* (1 × 108 CFU mL^−1^), and *T. harzianum* (1 × 10^5^ spores mL^−1^) per 30 g of sterilized carrier and mixed with the soybean seeds before sowing using a sticking material and spread away from the direct sun over a plastic sheet for a short time prior planting.

#### 4.3.2. Beneficial and Micro-Elements Nanoparticles

The nanoparticles of silicon dioxide (SiO_2_: 198 nm particle size) was provided by AL-Azhar University, Egypt, while the nanoparticles of zinc oxide (ZnO: <100 nm particle size) was purchased from Sigma-Aldrich (St. Louis, USA). Nano-elements (Si+Zn) were applied as a foliar application at 500 mg L^−1^ (250 mg L^−1^ for each element) at 20, 30, and 40 days after sowing (DAS), which was applied as a nutrient solution.

### 4.4. Traits Measurements

At 60 days after sowing, different plant samples were collected to determine the following variables: root length, nodules dry weight, the content of Na^+^ and K^+^ ions in the leaves, total chlorophyll, carotenoids, activity of antioxidant enzymes, malondialdehyde, total soluble sugars, proline, total free amino acids, and soluble proteins. In addition to leaf relative water content (LRWC), stomatal conductance, and electrolyte leakage (EL%).

#### 4.4.1. Exchangeable Sodium % (ESP)

An auger was used to collect soil samples from the surface of the soil down to a depth of 40 cm. Soil samples were air-dried and passed through a 2-mm mesh to determine the contents of Na^+^, Ca^2+^ and Mg^2+^ in paste extracts. The contents of Na^+^, Ca^2+^ and Mg^2+^ in paste extracts were determined using an Atomic Absorption Spectro-photometer (AAS 3300, Perkin Elmer Ltd., Beaconsfield, UK) to compute the soil sodium adsorption ratio (SAR). ESP was assessed according to the equation proposed by Seilsepour et al. [54]:

$$ESP=1.95+1.03\times \mathrm{SAR}\;\left(R^{2}=0.92\right)$$

#### 4.4.2. Root Length, Nodules Dry Weight and Leaves Area

Root samples were taken from each treatment type to estimate the root length (cm) and nodules dry weight (mg plant^−1^). The selected root samples were taken by soil sampling tube to 40 cm depth and sieving the soil through a 0.5 mm mesh. Nodules were detached from the roots, oven-dried at 70 °C and weighed. Total leaves area per plant (dm^2^) was recorded by Leaf Area Meter (LA-3000A, LI-COR Inc., Lincoln, NE, USA).

#### 4.4.3. Potassium and Sodium Concentration

Ten leaf samples were randomly collected from each experimental plot, washed with deionized water, and oven-dried for 48 h at 70 °C. Oven-dried samples (approximately 0.5 g each) were heated for 5 h in a mixture of nitric acid and perchloric acid (4:1, *v*/*v*) in the presence of glass beads. The digested plant material was sieved before being diluted with de-ionized water, based on Chapman and Pratt [55] method. Na^+^ and K^+^ concentrations were estimated by the use of an AAS 3300 Atomic Absorption Spectrophotometer (Perkin Elmer Ltd.).

#### 4.4.4. Total Chlorophylls and Carotenoids Concentration

Using the protocol described by Lichtenthaler [56], ten leaf samples were randomly taken from the top of the main stem in each treatment to determine total chlorophylls and carotenoids. Samples of leaf tissues (0.1 g each) were ground and extracted in 5 mL of acetone at an 80% concentration. The extracted sap was centrifuged for 10 min at 13,000× *g*, and the absorbance of the supernatant was measured at 663 nm, 645 nm, and 470 nm. Concentrations of total chlorophylls and carotenoids were calculated and expressed as mg g^−1^ FW.

#### 4.4.5. Osmo-protectants and Protein Concentrations

##### Total Soluble Sugars (TSS) 

Leaves fresh samples (0.5 g) were homogenized in 5 mL of 80% (*v*/*v*) ethanol and placed in a water bath at 80 °C for 30 min, followed by centrifugation at 10,000× *g* for 10 min. The pellets were subjected to two further cycles using the same extraction protocol by 80% ethanol. The supernatants were collected to measure the total soluble sugars concentration based on Hendrix [57] protocol, using spectrophotometer at 620 nm wavelength, based on a glucose standard curve. 

##### Proline

Concentrations of proline in 0.5 g leaf samples were determined using the methods described by Bates et al. [58]. Samples were homogenized in 10 mL of 3 % (m/v) aqueous sulfosalicylic acid and centrifuged. Each 2 mL of supernatant was combined with 2 mL of acid ninhydrin reagent (2 g ninhydrin in 30 mL glacial acetic acid and 20 mL 6 M phosphoric acid) and 2 mL of glacial acetic acid. After the reaction mixture was boiled at 100 °C for 1 h and then cooled in an ice bath for 10 min, the colour of the reaction mixture was extracted by adding 4 mL toluene and vortexed for 5 min. The absorbance of coloured toluene was read at 520 nm. Proline was estimated as μmol g^−1^ FW leaves.

##### Free Amino Acid (FAA) 

Total free amino acids were extracted from 0.5 g of fresh leaf samples after the homogenization with 80% ethanol and centrifugation at 8000× *g* for 15 min at 4 °C. Amino acids concentration was assayed in the supernatant using the ninhydrin reagent as described by Misra et al. [59]. The analysis was performed by the addition of ninhydrin reagent (16% ninhydrin dissolved in citrate buffer, pH 5.0 [26% citric acid, and 58% Na citrate]) to amino acid extract. The mixture was boiled for 20 min and then cooled to room temperature. Absorbance of the reaction was recorded at 570 nm. Concentrations of the total free amino acids in leaves samples were calculated from the standard curve of L-leucine and presented as mg g^−1^ FW. 

##### Total Soluble Protein (TSP)

The concentration of soluble protein in leaves samples were estimated by the Brilliant Blue G-250 reagent with bovine serum albumin (BSA) as a standard based on the method described by Bradford [60].

#### 4.4.6. Activity of Antioxidant Enzymes (CAT, POD and SOD)

Extracts of enzymatic antioxidant were made by freezing different leaves samples (1 g) in the liquid nitrogen to avoid proteolytic activity, then grinding with 5 mL of cold extraction buffer (0.1 M phosphate buffer, pH 7, containing 0.5 mM EDTA, and 2 % [*w*/*v*] PVP), and centrifuging for 20 min at 10,000× *g* to use the supernatant as enzyme extract [11].

The activity of catalase (CAT), peroxidase (POD) and superoxide dismutase (SOD), were estimated at 25 °C, using a UV-160 A spectrophotometer (Shimadzu, Kyoto, Japan). The specific activity of CAT, POD, and SOD were estimated based on the protocols described by Aebi [61], Vetter et al. [62], and Beauchamp and Fridovich [63], respectively. The specific activity of the antioxidant enzymes is expressed as Unit mg^−1^ protein. Briefly, CAT activity in the samples was estimated based on tracking the H_2_O_2_ fading at 240 nm due to the catalytic activity of CAT in the samples. The activity of POD in the samples was determined by combining 100 μL enzyme extract with 2.9 mL 50 mM phosphate-citrate buffer (pH 6.5), 0.03% H_2_O_2_, and 0.1% *ortho*-phenylenediamine in the assay mixture. At 430 nm, the change in absorbance was measured for 5 min. SOD activity was determined based on the inhibition in the photoreduction of the dye nitroblue tetrazolium (NBT) by superoxide-formed radicals. The reaction mixture (consists of 100 μL enzyme extract in 50 mM phosphate buffer, 13 mM methionine, 0.075 mM NBT, 0.10 mM EDTA and 0.002 mM riboflavin) was illuminated for 15 min in a light chamber. The absorbance of blue formazan formed dye was recorded directly at 550 nm in comparison to the sample free reaction mixture. The enzymatic unit of SOD has been defined as the amounts of the enzyme required to inhibit the reduction of chromogen by 50%.

#### 4.4.7. Oxidative Damage Status

##### Malondialdehyde (MDA)

The concentration of MDA in the leaves as an indicator for lipid peroxidation was performed according to the protocol described by Li et al. [64]. With a mortar and pestle, 0.5 g of fresh leaves was homogenized with 1 mL 0.1% (*w*/*v*) TCA solution. The mixture was centrifuged at 8000× *g* for 15 min at 4 °C. For each 1 mL supernatant, 4 mL of 0.5% thiobarbituric acid (TBA) in 10% TCA solution was added and incubated at 96 °C for 30 min. After cooling, the absorbance (A) was recorded at 450 nm, 532 nm, and 600 nm. The MDA concentration was expressed as nmol g^−1^ FW based on the extinction coefficient of TBA (1.53 mM^−1^cm^−1^).

##### Electrolyte Leakage Percentage (EL%) 

The protocol described by Bajji, et al. [65] was used to estimate EL%. Ten leaf discs were put in a 50 mL sealed tube including 20 mL distilled water and the electrical conductivity (EC_0_) was measured by a conductivity meter (CM 100, John Reid and Associates, Chicago). The tubes were incubated in a water bath at 45 °C for 30 min, and the electrical conductivity (EC_1_) was measured. The sample was heated at 100 °C for 10 min, and the electrical conductivity (EC_2_) was measured. The total leakage of inorganic ions was calculated according to the following equation: EL% = [(EC_1_ − EC_0_)/EC_2_] × 100.

#### 4.4.8. Plant Water Relations

##### Leaf Relative Water Content (LRWC)

For the estimation of LRWC, the method of Weatherley [66] was used. Ten leaf discs with 10 mm in diameter were punched with a borer from a set of leaves into a reweighed sealed vial. After the fresh weight had been obtained, the discs were floated for 24 h on distilled water in covered Petri dishes kept at a low light intensity and room temperature until full turgid. The discs were surface dried, returned to the same vial, and reweighed to obtain the turgid weight. Finally, the leaf discs were oven-dried at 80 °C to a constant weight and weighed again to obtain the dry weight. The LRWC on a percentage basis was calculated using the following equation:

$$\mathrm{LRWC}=\left(\frac{\mathrm{Fresh}\;\mathrm{weight}-\mathrm{Dry}\;\mathrm{weight}}{\mathrm{Turgid}\;\mathrm{weight}-\mathrm{Dry}\;\mathrm{weight}}\right)\times 100$$

##### Stomatal Conductance

Stomatal conductance of fully expanded top leaf was determined between 10:00 and 13:00 using porometer apparatus (Model AP4, Delta-T Devices Ltd., Burwell, UK) and expressed as mmol H_2_O m^−2^ s^−1^.

#### 4.4.9. Seed Yield Related Traits and Nutritional Quality

At maturity, the aboveground parts of soybean plants in each plot were harvested and were used as shoot samples for calculating the number of pods plant^−1^. Soybean seeds were separated from their pods to measure 100-seed weight (g) and seed yield (kg ha^−1^). The protein content of soybean seeds was estimated via the micro-Kjeldahl method based on N content. Nitrogen was evaluated according to the method illustrated in A.O.A.C. [67], and the protein content was obtained by multiplying the nitrogen content by a conventional coefficient of 6.25. Total carbohydrate contents were extracted from dry powdered soybean seeds and estimated colorimetrically by the phenol-sulphuric acid method as described by Sadasivam and Manickam [68].

### 4.5. Statistical Analysis

The data of the two-year split-plot experiment were statistically analyzed separately according to the analysis of variance (ANOVA) procedure, using CoStat software (Package 6.45, CoHort, Monterey, CA, USA). The differences between the means were compared at *p* < 0.05 using DMRT as posthoc pairwise comparisons [69]. Data are presented as means ± SD. The comparisons of parallel coordinates were performed by JMP 16 (SAS Institute Inc., Cary, NC, USA).

## 5. Conclusions

Water stress and soil salinity together greatly lessen the plant growth and crop productivity, which effect on sensitive to moderate tolerant plants like soybean plant. Nevertheless, the coupled application of seed inoculation with PGPMs (*Bradyrhizobium japonicum* (USDA 110) and *Trichoderma harzianum*) and foliar application of Si-ZnNPs significantly mitigated the detrimental effects of water stress and soil salinity on soybean growth and productivity. Irrespective of water application duration (IW_0_, IW_1,_ and IW_2_), the co-application treatment of PGPMs and Si-ZnNPs caused the highest seed quality (i.e., protein and carbohydrate content) and highest yield-related traits (number of pods plant^−1^ and 100-seed weight) of soybean plants grown under salt-affected soil in comparison to sole application of PGPMs or Si-ZnNPs and/or control plants. Such improvements were attributed to deeper root length, heavier nodules dry weight, higher leaf K^+^, further leaf soluble protein content, total soluble sugars, and antioxidant enzymes activity, as well as more augmented of physiological parameters (relative water content and stomatal conductance) while declined of leaf Na^+^, electrolyte leakage, proline content, and free amino acids, as well as ESP, were attained when the coupled treatment was added compared to the sole treatment of PGPMs or Si-ZnNPs under longer irrigation intervals (IW_1_ and IW_2_). Our study proved that the coupled application of PGPMs and Si-ZnNPs has multiple benefits for agricultural sustainability, especially in arid and semi-arid zones.

## Figures and Tables

**Table 1:** Characteristics of yield and seed nutritional quality of soybean plant treated with PGPMs and/or Si-ZnNPs, and grown in salt-affected soil with different levels of watering intervals (**IW_0_**; 11 days, **IW_1_**; 15 days, and **IW_2_**; 19 days) during 2019 and 2020 growing seasons.

Season	Irrigation	Treatments	Pods Plant^−1^ (No.)	100-Seed (g)	Seed Yield (t ha^−1^)	Protein Seed^−1^ (%)	Carb. Seed^−1^ (%)
2019	IW_0_	Control	82.4 ± 5.2 de	16.1 ± 0.8 c	1.7 ± 0.08 e	28.1 ± 1.0 cd	20.2 ± 1.2 c
		PGPMs	99.7 ± 4.7 c	17.5 ± 1.1 b	2.0 ± 0.14 c	33.1 ± 1.1 b	23.4 ± 1.2 b
		Si-ZnNPs	105.6 ± 4.0 b	18.0 ± 1.0 b	2.1 ± 0.11 b	35.1 ± 1.4 b	26.0 ± 1.0 a
		PGPMs + Si-ZnNPs	115.3 ± 4.1 a	19.4 ± 1.3 a	2.3 ± 0.12 a	38.3 ± 1.6 a	27.9 ± 1.0 a
	IW_1_	Control	64.6 ± 3.8 h	13.7 ± 0.8 eg	1.3 ± 0.07 h	18.4 ± 1.4 g	15.9 ± 0.9 ef
		PGPMs	79.6 ± 3.2 e	14.5 ± 0.5 de	1.5 ± 0.09 f	23.8 ± 1.2 e	18.7 ± 0.8 cd
		Si-ZnNPs	86.4 ± 3.0 d	15.2 ± 1.0 cd	1.6 ± 0.11 e	26.3 ± 1.3 d	19.9 ± 1.1 c
		PGPMs + Si-ZnNPs	96.1 ± 4.6 c	16.3 ± 1.3 c	1.8 ± 0.09 d	30.1 ± 1.5 c	22.2 ± 1.1 b
	IW_2_	Control	57.7 ± 2.5 i	11.9 ± 0.7 h	0.9 ± 0.07 k	11.3 ± 1.1 i	11.4 ± 1.0 h
		PGPMs	70.4 ± 4.3 g	12.5 ± 0.9 gh	1.1 ± 0.07 j	14.4 ± 1.3 h	13.1 ± 0.9 gh
		Si-ZnNPs	73.2 ± 3.3 fg	13.2 ± 1.1 fg	1.2 ± 0.06 i	16.7 ± 0.9 gh	14.4 ± 0.6 fg
		PGPMs + Si-ZnNPs	76.9 ± 4.4 ef	13.8 ± 1.1 ef	1.4 ± 0.08 g	20.9 ± 1.91 f	17.2 ± 1.5 de
2020	IW_0_	Control	84.0 ± 4.1 e	16.3 ± 0.9 cd	1.8 ± 0.09 d	29.5 ± 0.9 de	21.3 ± 1.9 cd
		PGPMs	102.3 ± 2.4 c	17.3 ± 0.9 bc	2.1 ± 0.07 c	33.9 ± 1.9 c	24.7 ± 1.1 b
		Si-ZnNPs	107.8 ± 3.5 b	18.2 ± 1.1 b	2.2 ± 0.05 b	36.5 ± 1.1 b	27.2 ± 0.9 a
		PGPMs + Si-ZnNPs	118.3 ± 3.9 a	20.5 ± 0.9 a	2.5 ± 0.11 a	39.7 ± 1.2 a	28.8 ± 1.3 a
	IW_1_	Control	68.9 ± 2.7 f	14.9 ± 0.9 ef	1.3 ± 0.09 f	19.6 ± 1.4 h	16.8 ± 1.1 fg
		PGPMs	83.3 ± 2.3 e	15.5 ± 0.9 df	1.6 ± 0.09 e	25.2 ± 1.4 f	19.4 ± 1.2 de
		Si-ZnNPs	90.4 ± 3.3 d	16.1 ± 0.9 ce	1.8 ± 0.08 d	27.7 ± 1.2 e	20.6 ± 1.0 cd
		PGPMs + Si-ZnNPs	102.2 ± 4.6 c	16.9 ± 0.8 c	2.0 ± 0.11 c	31.1 ± 1.7 d	22.6 ± 1.1 c
	IW_2_	Control	60.5 ± 4.5 g	13.1 ± 1.1 g	1.0 ± 0.08 g	12.5 ± 0.9 j	12.5 ± 1.1 i
		PGPMs	71.0 ± 3.1 f	14.3 ± 1.0 fg	1.3 ± 0.08 f	15.6 ± 1.1 i	14.3 ± 0.7 hi
		Si-ZnNPs	73.5 ± 3.7 f	14.6 ± 1.0 f	1.3 ± 0.07 f	18.4 ± 1.2 h	15.7 ± 0.9 gh
		PGPMs + Si-ZnNPs	83.3 ± 5.4 e	15.4 ± 1.0 df	1.6 ± 0.06 e	22.2 ± 2.1 g	18.4 ± 1.6 ef
*F* test	Deficit irrigation		***	**	***	***	***
	Soil and foliar treatments		***	***	***	***	***
	Irrigation X Treatments		*	ns	**	ns	ns

Means followed by different lower-case letters indicate significant differences among treatments according to Duncan’s Multiple Range Test (*P* < 0.05). Values are presented as means ± standard deviation (SD). ***^,^ **^,^ *, and ns denote significance at *P* < 0.001, *P* < 0.01, and non-significant, respectively. **PGPMs**: Plant growth-promoting microbes, **NP**: Nano particles, **Carb**: Carbohydrate content, **IW**: Interval watering.

## References

[1] Munns R., Gilliham M. (2015). Salinity tolerance of crops—What is the cost?. New Phytol..

[2] Negrão S., Schmöckel S.M., Tester M. (2017). Evaluating physiological responses of plants to salinity stress. Ann. Bot.

[3] Parihar P., Singh S., Singh R., Singh V.P., Prasad S.M. (2014). Effect of salinity stress on plants and its tolerance strategies: A review. Environ. Sci. Pollut. Res..

[4] Munns R., Tester M. (2008). Mechanisms of salinity tolerance. Annu. Rev. Plant. Biol..

[5] Hafez E.M., Kheir A.M.S., Badawy S.A., Rashwan E., Farig M., Osman H.S. (2020). Differences in physiological and biochemical attributes of wheat in response to single and combined salicylic acid and biochar subjected to limited water irrigation in saline sodic soil. Plants.

[6] Parida A.K., Das A.B. (2005). Salt tolerance and salinity effects on plants: A review. Ecotoxic. Environ. Safety.

[7] Salim B.B.M., Hikal M.S., Osman H.S. (2019). Ameliorating the deleterious effects of saline water on the antioxidants defense system and yield of eggplant using foliar application of zinc sulphate. Ann. Agric. Sci..

[8] Osman H.S., Salim B.B. (2016). Improving yield and quality of kohlrabi stems growing under NaCl salinity using foliar application of urea and seaweed extract. J. Hort. Sci. Ornament. Plant..

[9] Osman H.S., Salim B.B.M. (2016). Enhancing antioxidants defense system of snap bean under NaCl salinity using foliar application of salicylic acid, spermidine and glycine betaine. Am. Eurasian J. Agric. Environ. Sci..

[10] Wang B., Zhang J., Pei D., Yu L. (2021). Combined effects of water stress and salinity on growth, physiological, and biochemical traits in two walnut genotypes. Physiol. Plant..

[11] Osman H.S. (2015). Enhancing antioxidant–yield relationship of pea plant under drought at different growth stages by exogenously applied glycine betaine and proline. Ann. Agric. Sci..

[12] Hafez E., Omara A.E.D., Ahmed A. (2019). The coupling effects of plant growth promoting rhizobacteria and salicylic acid on physiological modifications, yield traits, and productivity of wheat under water deficient conditions. Agronomy.

[13] Munns R. (2002). Comparative physiology of salt and water stress. Plant. Cell Environ..

[14] Sahin U., Ekinci M., Ors S., Turan M., Yildiz S., Yildirim E. (2018). Effects of individual and combined effects of salinity and drought on physiological, nutritional and biochemical properties of cabbage (*Brassica oleracea* var. *capitata*). Sci. Hortic..

[15] Araújo S.S., Beebe S., Crespi M., Delbreil B., González E.M., Gruber V., Lejeune-Henaut I., Link W., Monteros M.J., Prats E. (2015). Abiotic stress responses in legumes: Strategies used to cope with environmental challenges. Crit. Rev. Plant. Sci..

[16] Pagano M.C., Miransari M., Miransari M. (2016). The importance of soybean production worldwide. Abiotic and Biotic Stresses in Soybean Production.

[17] Han H.S., Lee K.D. (2005). Physiological responses of soybean-inoculation of *Bradyrhizobium japonicum* with PGPR in saline soil conditions. Res. J. Agric. Biol. Sci..

[18] Maas E.V., Grattan S.R., Skaggs R.W., van Schilfgaarde J. (1999). Crop yields as affected by salinity. Agricultural Drainage.

[19] Ku Y.-S., Au-Yeung W.-K., Yung Y.-L., Li M.-W., Wen C.-Q., Liu X., Lam H.-M., Board J.E. (2013). Drought stress and tolerance in soybean. A Comprehensive Survey of International Soybean Research—Genetics, Physiology, Agronomy and Nitrogen Relationships.

[20] Liu F., Jensen C.R., Andersen M.N. (2004). Drought stress effect on carbohydrate concentration in soybean leaves and pods during early reproductive development: Its implication in altering pod set. Field Crops Res..

[21] Hafez E., Farig M. (2019). Efficacy of salicylic acid as a cofactor for ameliorating effects of water stress and enhancing wheat yield and water use efficiency in saline soil. Int. J. Plant. Prod..

[22] Hafez E.M., Alsohim A.S., Farig M., Omara A.E.-D., Rashwan E., Kamara M.M. (2019). Synergistic effect of biochar and plant growth promoting rhizobacteria on alleviation of water deficit in rice plants under salt-affected soil. Agronomy.

[23] Hafez E.M., Omara A.E.D., Alhumaydhi F.A., El-Esawi M.A. (2020). Minimizing hazard impacts of soil salinity and water stress on wheat plants by soil application of vermicompost and biochar. Physiol. Plant..

[24] Hafez E.M., Osman H.S., Gowayed S.M., Okasha S.A., Omara A.E.-D., Sami R., Abd El-Monem A.M., Abd El-Razek U.A. (2021). Minimizing the adversely impacts of water deficit and soil salinity on maize growth and productivity in response to the application of plant growth-promoting rhizobacteria and silica nanoparticles. Agronomy.

[25] Hafez E.M., Osman H.S., El-Razek U.A.A., Elbagory M., Omara A.E.-D., Eid M.A., Gowayed S.M. (2021). Foliar-applied potassium silicate coupled with plant growth-promoting rhizobacteria improves growth, physiology, nutrient uptake and productivity of faba bean (*Vicia faba* L.) irrigated with saline water in salt-affected soil. Plants.

[26] Mendes R., Garbeva P., Raaijmakers J.M. (2013). The rhizosphere microbiome: Significance of plant beneficial, plant pathogenic, and human pathogenic microorganisms. FEMS Microbiol. Rev..

[27] Olanrewaju O.S., Glick B.R., Babalola O.O. (2017). Mechanisms of action of plant growth promoting bacteria. World J. Microbiol. Biotechnol..

[28] Ma Y., Dias M.C., Freitas H. (2020). Drought and salinity stress responses and microbe-induced tolerance in plants. Front. Plant. Sci..

[29] Kumar A., Verma J.P. (2018). Does plant—Microbe interaction confer stress tolerance in plants: A review?. Microbiol. Res..

[30] Egamberdieva D., Wirth S.J., Alqarawi A.A., Abd Allah E.F., Hashem A. (2017). Phytohormones and beneficial microbes: Essential components for plants to balance stress and fitness. Front. Microbiol..

[31] Solyman S.N., Abdel-Monem M., Abou-Taleb K., Osman H.S., El-Sharkawy R.M. (2019). Production of plant growth regulators by some fungi isolated under salt stress. South Asian J. Res. Microbiol..

[32] Masciarelli O., Llanes A., Luna V. (2014). A new PGPR co-inoculated with *Bradyrhizobium japonicum* enhances soybean nodulation. Microbiol. Res..

[33] Egamberdieva D., Wirth S., Jabborova D., Räsänen L.A., Liao H. (2017). Coordination between *Bradyrhizobium* and *Pseudomonas* alleviates salt stress in soybean through altering root system architecture. J. Plant. Interact..

[34] Hermosa R., Viterbo A., Chet I., Monte E. (2012). Plant-beneficial effects of *Trichoderma* and of its genes. Microbiology.

[35] Mona S.A., Hashem A., Abd_Allah E.F., Alqarawi A.A., Soliman D.W.K., Wirth S., Egamberdieva D. (2017). Increased resistance of drought by *Trichoderma harzianum* fungal treatment correlates with increased secondary metabolites and proline content. J. Integrat. Agric..

[36] Marra R., Lombardi N., d’Errico G., Troisi J., Scala G., Vinale F., Woo S.L., Bonanomi G., Lorito M. (2019). Application of *Trichoderma* strains and metabolites enhances soybean productivity and nutrient content. J. Agric. Food Chemist..

[37] Broadley M., Brown P., Cakmak I., Rengel Z., Zhao F., Marschner P. (2012). Function of nutrients: Micronutrients. Marschner’s Mineral Nutrition of Higher Plants.

[38] Kheir A.M.S., Abouelsoud H.M., Hafez E.M., Ali O.A.M. (2019). Integrated effect of nano-Zn, nano-Si, and drainage using crop straw–filled ditches on saline sodic soil properties and rice productivity. Arab. J. Geosci..

[39] Osman H.S., Abd El-Gawad H.G. (2013). Impact of stimulators of amylase activity (GA_3_, CaCl_2_) and protein synthesis (ZnSO_4_) on yield, quality and reducing seed abortion of pea plant. Res. J. Agric. Biol. Sci..

[40] Batista P.F., Müller C., Merchant A., Fuentes D., Silva-Filho R.d.O., da Silva F.B., Costa A.C. (2020). Biochemical and physiological impacts of zinc sulphate, potassium phosphite and hydrogen sulphide in mitigating stress conditions in soybean. Physiol. Plant..

[41] Youssif N.E.E., Osman H.S.M., Salama Y.A.M., Zaghlool S.A.M. (2018). Effect of rice straw and applications of potassium silicate, potassium humate and seaweed extract on growth and some macronutrients of sweet pepper plants under irrigation deficit. Arab Univ. J. Agric. Sci..

[42] Seleiman M.F., Refay Y., Al-Suhaibani N., Al-Ashkar I., El-Hendawy S., Hafez E.M. (2019). Integrative effects of rice-straw biochar and silicon on oil and seed quality, yield and physiological traits of *Helianthus annuus* L. grown under water deficit stress. Agronomy.

[43] Yeo A.R., Flowers S.A., Rao G., Welfare K., Senanayake N., Flowers T.J. (1999). Silicon reduces sodium uptake in rice (*Oryza sativa* L.) in saline conditions and this is accounted for by a reduction in the transpirational bypass flow. Plant. Cell Environ..

[44] Liu R., Lal R. (2015). Potentials of engineered nanoparticles as fertilizers for increasing agronomic productions. Sci. Total Environ..

[45] Boiero L., Perrig D., Masciarelli O., Penna C., Cassán F., Luna V. (2007). Phytohormone production by three strains of *Bradyrhizobium japonicum* and possible physiological and technological implications. Appl. Microbiol. Biotech..

[46] Oliveira C.M., Almeida N.O., da Rocha M.R., Rezende M.H., Carneiro R.G.d.S., Ulhoa C.J. (2020). Anatomical changes induced by isolates of *Trichoderma* spp. in soybean plants. PLoS ONE.

[47] Pal V., Singh G., Dhaliwal S.S. (2020). Symbiotic parameters, growth, productivity and profitability of chickpea as influenced by zinc sulphate and urea application. J. Soil Sci. Plant. Nutr..

[48] Tripathi P., Na C.-I., Kim Y. (2021). Effect of silicon fertilizer treatment on nodule formation and yield in soybean (*Glycine max* L.). Eur. J. Agron..

[49] Osman H.S., Salim B.B.M. (2016). Influence of exogenous application of some phytoprotectants on growth, yield and pod quality of snap bean under NaCl salinity. Ann. Agric. Sci..

[50] McKeague J.A. (1978). Manual on Soil Sampling and Methods of Analysis.

[51] Mclean E.O., Page A.L. (1983). Soil pH and Lime Requirement. Methods of Soil Analysis.

[52] El-Nahrawy S., Elbagory M., Omara A.E.-D. (2020). Biocompatibility effect of *Bradyrhizobium japonicum* and *Trichoderma* strains on growth, nodulation and physiological traits of soybean (*Glycine max* l.) under water deficit conditions. J. Adv. Microbiol..

[53] Atlas R.M. (2010). Handbook of Microbiological Media.

[54] Seilsepour M., Rashidi M., Khabbaz B.G. (2009). Prediction of soil exchangeable sodium percentage based on soil sodium adsorption ratio. Am. Eurasian J. Agric. Environ. Sci..

[55] Chapman H.D., Pratt P.F. (1962). Methods of Analysis for Soils, Plants and Waters. Soil Sci..

[56] Lichtenthaler H.K. (1987). Chlorophylls and carotenoids: Pigments of photosynthetic biomembranes. Methods in Enzymology.

[57] Hendrix D.L. (1993). Rapid extraction and analysis of nonstructural carbohydrates in plant tissues. Crop. Sci..

[58] Bates L.S., Waldren R.P., Teare I.D. (1973). Rapid determination of free proline for water-stress studies. Plant. Soil.

[59] Misra P.S., Mertz E.T., Glover D.V. (1975). Studies on corn proteins. VIII. Free amino acid content of opaque-2 double mutants. Cereal Chem..

[60] Bradford M.M. (1976). A rapid and sensitive method for the quantitation of microgram quantities of protein utilizing the principle of protein-dye binding. Analyt. Biochem..

[61] Aebi H. (1984). Catalase in vitro. Methods in Enzymology.

[62] Vetter J.L., Steinberg M.P., Nelson A.I. (1958). Enzyme assay, quantitative determination of peroxidase in sweet corn. J. Agric. Food Chemist..

[63] Beauchamp C., Fridovich I. (1971). Superoxide dismutase: Improved assays and an assay applicable to acrylamide gels. Analyt. Biochem..

[64] Li Q.-T., Yeo M.H., Tan B.K. (2000). Lipid peroxidation in small and large phospholipid unilamellar vesicles induced by water-soluble free radical sources. Biochem. Biophysic. Res. Communic..

[65] Bajji M., Kinet J.-M., Lutts S. (2002). The use of the electrolyte leakage method for assessing cell membrane stability as a water stress tolerance test in durum wheat. Plant. Growth Regul..

[66] Weatherley P.E. (1950). Studies in the water relations of the cotton plant. I. The field measurement of water deficits in leaves. New Phytol..

[67] A.O.A.C (2005). Official Methods of Analysis of AOAC International.

[68] Sadasivam S., Manickam A. (2010). Biochemical Methods.

[69] Duncan D.B. (1955). Multiple range and multiple F tests. Biometrics.

